# Contraceptive stockouts in Western Kenya: a mixed-methods mystery client study

**DOI:** 10.1186/s12913-023-09047-w

**Published:** 2023-01-24

**Authors:** Katherine Tumlinson, Laura E. Britton, Emilia Goland, Stephanie Chung, Brooke W. Bullington, Caitlin R. Williams, Debborah Muthoki Wambua, Dickens Otieno Onyango, Leigh Senderowicz

**Affiliations:** 1 Department of Maternal and Child Health, Gillings School of Global Public Health, University of North Carolina at Chapel Hill, 170 Rosenau Hall, CB #7400, 135 Dauer Drive, Chapel Hill, NC 27599-7400 USA; 2 Carolina Population Center, University of North Carolina at Chapel Hill, Chapel Hill, USA; 3 Columbia University School of Nursing, New York City, USA; 4 Department of Epidemiology, Gillings School of Global Public Health, University of North Carolina at Chapel Hill, Chapel Hill, USA; 5 Department of Mother and Child Health, Institute for Clinical Effectiveness and Health Policy (IECS), Buenos Aires, Argentina; 6 Innovations for Poverty Action-Kenya (IPA-K), Nairobi, Kenya; 7 Kisumu County Department of Health, Kisumu, Kenya; 8 Julius Global Health, Julius Centre for Health Sciences and Primary Care, University Medical Centre, Utrecht, Netherlands; 9 Department of Obstetrics and Gynecology, School of Medicine and Public Health, University of Wisconsin, Madison, WI USA

**Keywords:** Family planning, Reproductive health, Health services, Contraceptive stockouts, Contraceptive supply

## Abstract

**Background:**

The prevalence of modern contraception use is higher in Kenya than in most countries in Sub-Saharan Africa. The uptake has however slowed down in recent years, which, among other factors, has been attributed to challenges in the supply chain and increasing stockouts of family planning commodities. Research on the frequency of contraceptive stockouts and its consequences for women in Kenya is still limited and mainly based on facility audits.

**Methods:**

This study employs a set of methods that includes mystery clients, focus group discussions, key informant interviews, and journey mapping workshops. Using this multi-method approach, we aim to quantify the frequency of method denial resulting from contraceptive stockout and describe the impact of stockouts on the lived experiences of women seeking contraception in Western Kenya.

**Results:**

Contraceptives were found to be out of stock in 19% of visits made to health facilities by mystery clients, with all contraceptive methods stocked out in 9% of visits. Women experienced stockouts as a sizeable barrier to accessing their preferred method of contraception and a reason for taking up non-preferred methods, which has dire consequences for heath, autonomy, and the ability to prevent unintended pregnancy. Reasons for contraceptive stockouts are many and complex, and often linked to challenges in the supply chain – including inefficient planning, procurement, and distribution of family planning commodities.

**Conclusions:**

Contraceptive stockouts are frequent and negatively impact patients, providers, and communities. Based on the findings of this study, the authors identify areas where funding and sustained action have the potential to ameliorate the frequency and severity of contraceptive stockouts, including more regular deliveries, in-person data collection, and use of data for forecasting, and point to areas where further research is needed.

## Background

Recent estimates have shown that 218 million women of reproductive age (15–49) in low and middle-income countries have an unmet need for modern contraception – meaning that they do not wish to become pregnant but are not using contraceptives. A majority of these women live in Sub-Saharan Africa [1]. To meet an increased demand for modern contraception, supply chains need to be strengthened to avoid stockouts caused by factors such as transportation delays or procurement failures [2]. Stockouts, defined as the lack of available contraceptive commodities in locations such as healthcare facilities or pharmacies, where they are expected to be on-hand [3], can lead to a woman being denied the right to access a method of her choice, increasing the risk for contraceptive discontinuation and unwanted pregnancy [4]. Studies have shown that contraceptive use is greater when more methods are available [5], and women are willing to travel further to access facilities where there are no stockouts [6].

The magnitude and impact of modern contraception stockouts in Sub-Saharan Africa has so far received little attention in the scientific literature, and estimates vary by method of measurement [7]. While analysis of data from Demographic and Health Surveys (DHS) has shown that few women with unmet need for contraception state that they lack access [8], data from recent rounds of Performance Monitoring and Accountability (PMA) 2020 show that 46–67% of facilities in surveyed countries in Sub-Saharan Africa reported a stockout of at least one family planning method in the past three months [9]. The frequency of stockouts varies across geographic locations, types of facilities, and method type. While several studies have found stockouts to be more frequent in public sector facilities compared to private or not-for-profit [10, 11], others have found the opposite pattern [12]. Long-acting reversible contraceptives have also been found to be out of stock more often than other contraceptive methods [10, 11, 13].

The prevalence of modern contraception use in Kenya is higher than in most other Sub-Saharan African countries, but progress in uptake has slowed down in recent years, and many facilities are facing stockouts [14, 15]. A mapping of supply chains in Kenya published in 2021, showed that stockouts of family planning commodities have increased in the last 3 years [15, 16]. While the mechanisms behind the increase in stockouts in Kenya have not been researched in depth, many attribute increasing stockouts to factors such as the decentralization of government from the national to the county level following the 2010 Constitution and reduced domestic financing for contraceptives [16], as well as growing contraceptive demands and strikes by health workers, which in turn might have affected both procurement and distribution of commodities [14].

Employing data nested within a larger parent study designed to identify barriers to person-centered contraceptive care, we capitalize on the opportunity to assess stockouts via a unique methodological approach. Stockouts are typically measured via facility audits conducted in collaboration with a facility manager, who is typically given advance notice and may increase efforts to obtain supplies for the purposes of performing well on the audit. In contrast, the parent study employed mystery clients to assess facility-level quality, and, from these data, we can measure how often family planning clients experience stockouts as a barrier to obtaining their preferred method, or any method at all. The objective of this study is to quantify the frequency of method denial resulting from contraceptive stockout in Western Kenya. Our secondary aims are to qualitatively describe the impact of this facility-level barrier on the lived experiences of women seeking contraception in Western Kenya and to source locally grounded solutions.

## Methods

This study is a secondary data analysis of a larger parent study focused on identifying facility-level barriers to contraceptive uptake in Western Kenya. The parent study included a variety of data collection tools including mystery clients, focus group discussions, key informant interviews, and journey mapping workshops. We employ these data to measure the frequency and impact of contraceptive stockouts in Western Kenya – a region comprised of ten counties formerly known as Western and Nyanza province. Below, we provide a brief overview of data collection methods. An in-depth explanation of data collection methods, including sampling design and mystery client selection, can be found elsewhere [17].

### Sample of public healthcare facilities

We purposively selected five (of ten total) counties within Western Kenya that collectively cover areas inhabited by the four main tribes of the region. Within the five counties, we selected 60 public-sector facilities at random, from a total of 576 total public facilities in these countries. Prior to sampling, we first stratified by county and facility type.

### Mystery client methodology

We estimate the frequency of method denial resulting from contraceptive stockout using data from mystery client visits. Mystery clients are ‘under-cover’ data collectors who interact with providers and collect data on services received [18]. We deployed 15 mystery clients to 60 randomly selected public-sector facilities in Western Kenya, first stratifying by facility type: 1. dispensaries (*n* = 30); 2. health centers (*n* = 15); and 3. sub-county hospitals (*n* = 15). Mystery clients arrived at their assigned facility at 8:30 am and presented as new family planning clients. They used their true demographic profiles; ages ranged from 21 to 37 years, parity ranged from zero to two children, and seven were married. Each facility received three mystery client visits, resulting in a total of 180 visits, and the interval between each visit was approximately 12 days. We assigned each mystery client a ‘preferred’ method: three were assigned the intrauterine device (IUD) and four each were assigned the pill, injectable, and implant. Public-sector facilities are expected to stock all four of the methods sought by our mystery clients. Mystery clients recorded their observations using a brief electronic questionnaire within 30 minutes of their facility visit, which included questions on whether they were offered their preferred method and, if not, reasons for method denial. Data on method denial as a result of stockout are calculated as percentages, with the number of times a mystery client was denied contraception due to stockout in the numerator and the total number of visits (*n* = 180) in the denominator. We used Pearson’s chi-square tests to explore whether stockout of the preferred method, or all methods, varied by facility type or location (urban versus rural). Mystery client data were managed in Stata/SE 16.1.

### Qualitative methodology

We conducted eight focus group discussions (FGDs) containing six to eight participants each, ranging from ages 18 to 46. Community health volunteers identified and approached potential FGD participants and sought their permission to be contacted by study team members. FGDs participants were stratified as current or former family planning clients, urban or rural, and by county. We further conducted 19 healthcare sector key informant interviews (KIIs), with informants selected using a snowball sampling technique that began with the Head of Reproductive Health in select counties. Key informants were purposively selected, such that senior personnel from both public and private sector facilities were included. Key informants included senior staff from public and private-sector healthcare facilities and non-governmental public health organizations, as well as government officials. All FGDs and KIIs were conducted by trained enumerators using semi-structured questionnaires designed to explore facility-level barriers to family planning. FGD and KII data were assessed using a qualitative description approach to conduct conventional content analysis of all qualitative transcripts [19].

Finally, data from mystery clients, FGDs, and KIIs were synthesized into a client journey map (CJM) and a provider journey map (PJM). Journey maps allow visual depiction of the process via which women seek family planning as well as the process through which providers offer such services [20]. We validated the content of the journey maps using client and provider workshops, in which participants were invited to provide feedback on the maps.

We used professional transcription and English language translation (where needed) of all audio recordings of the FGDs, KII, and journey mapping workshops. An American and a Kenyan member of the research team - both with qualitative methods training – analyzed transcripts using a qualitative description approach to conduct conventional content analysis, reading each transcript holistically and generating a detailed codebook. We managed all qualitative data with NVivo 11.0 (QSR International).

Focus group and workshop participants, as well as key informants, provided written consent to participate. Ethical approval for the study protocol was provided by the University of North Carolina at Chapel Hill and the Kenya Medical Research Institute (KEMRI). Both IRBs necessarily waived informed consent for the mystery client portion of the study to avoid interfering with the study design.

## Results

### Frequency of stockouts

As shown in Table 1, in 19% of mystery client visits (35 out of 180 visits), the mystery client was told by their provider that their preferred method was out of stock. In 9% of visits (16 out of 180 visits), all modern methods were reportedly out of stock. In nearly half (49%) of those visits for which a preferred method was out of stock, the preferred method was the injectable. The 16 visits with all methods out of stock are a subset of the 35 visits in which the preferred method was stocked out. There was no relationship between the facility type or location (urban versus rural) and whether one or all methods were out of stock (data not shown).

**Table 1:** Frequency and characteristics of method stockouts during 180 mystery client visits to 60 public-sector healthcare facilities in Western Kenya, 2018–2019

	Total observations	Percentage
**Client’s preferred contraceptive method was out of stock**	*N* = 180	
Yes	35	19%
**All contraceptive methods were out of stock**	*N* = 180	
Yes	16	9%
**Mix of preferred methods that were out of stock**	*N* = 35	
Pills (Daily)	6	17%
Injectable	17	49%
Intrauterine Device	9	26%
Implant	3	9%

### Impact of stockouts on patients

Contraceptive stockout was perceived as one of the most salient and impactful reasons in our FGDs and KIIs for method denial, for both new users and ongoing users.

If you were to get an injection today, you go they tell you there’s no injection. You can really wait for a long time, so some people just go to the chemist. Because sometimes you can even wait for three days. You’re told there is no injection. You are told next week, next week, next week (Discontinued user, rural Kisumu County).

When their desired method was not available, some women accepted methods they did not particularly want:

The reason I stopped going to the public hospital is because I used the injection, and when I go to the hospital, I’m told it’s not available. So as [another participant] said, they convince you. The truth is that they convinced me until they gave me a five-year plan (method) (Current user, urban Kisumu County).

Other women responded to stockouts by patronizing the chemist or other private facilities, a work-around which was inaccessible for low-income women. Some women travelled to public facilities that were farther away, which was hard for women engaging in covert contraceptive use or with limited resources for travel. Others went without contraception.

### Impact of stockouts on providers

An insufficiently stocked healthcare facility also impacted providers by diminishing their vocational motivation.

To work effectively as a nurse I have to have commodities, I have to have the clean environment, I have all that plus wages because whether I am given a hundred million shillings as a salary and (in) my working environment the systems fail, I don’t think I’ll be motivated … this nurse doesn’t have soap to wash hands, she doesn’t have a broom to sweep the environment, so that is why I blanket said systems failure, you see systems … systems strengthening should go a long way with wages (Senior Government Official).

Irregular commodity supplies diminish trust and esteem (or high regard) in the healthcare system among community members who no longer expect reliable supply of contraceptives: ‘People tell each other (hakuna madawa huko) ‘there are no drugs there’ so nobody will come, then in the long run you will find mothers with pregnancy, unwanted pregnancies’ (Senior Government Official). Key informants were aware that stockouts of supplies created opportunities for informal fees, including requests to cover costs of equipment associated with insertions, syringes, or iodine. Providers might also ask for payments to go purchase the medications elsewhere themselves. Thus, stockouts were a barrier to motivated providers performing their role, cultivating a positive patient-provider relationship, and doing the work they desired to do: ‘Let the stakeholders provide adequate equipment, adequate space, and adequate supplies so that we are able to offer adequate service to our clients’ (Private Sector Facility Director).

### Strategies and solutions to reduce contraceptive stockouts

FGDs and KIIs conveyed participant attitudes and perceptions of Kenya’s supply chain as well as strategies for reform, although KII participants also noted the limitations of suggested strategies and the need to address such limitation to make these scalable solutions. Key informants in the healthcare system perceived stockouts were one of many barriers to family planning provision, such that it was necessary to address but ‘there is no one effective [solution] because all these [barriers] have to be addressed simultaneously’ (Senior Government Official). With these caveats, participants suggested solutions that coalesced around three primary areas: supply chain reform, documentation, and redistribution, as described below.

#### Supply chain reform

Contraceptive stockout was largely viewed as a policy problem requiring policy solutions, namely reform of the supply chain. Women in the community, as well as key informants in the healthcare system, conceptualize the government as responsible for maintaining the supply chain: ‘Because if the government could have brought the medicine, we could not have gotten problems’ (Current user, rural Busia County). Stock of ancillary supplies was also important, including working autoclaves, speculums, intrauterine device (IUD) insertion kits, pregnancy tests, and teaching materials for patient education. Notably, availability of complete IUD insertion kits made ordering easier. Some participants objected to the norm that clients were instructed to go purchase supplies they needed, with an example from maternity that highlighted how lacking a stock of ancillary supplies was a barrier to care:

You get to a facility with a woman in labor and you are told go and buy cotton wool, go and buy chlorhexidine. I was in a facility, and I heard them tell one of the women to go and buy chlorhexidine. In the first place this woman could not even pronounce the word chlorhexidine (Private Sector/NGO High-level Staff).

A key informant from nongovernmental organizations (NGOs) stated, ‘The bigger solution actually lies with the government.’ (Private Sector/NGO High-level Staff). Stock instability arising from incomplete and delayed order fulfilment was attributed to KEMSA (Kenya Medical Supplies Authority), the organization which ran the distribution program for family planning that was free to clients: providers described how it was challenging to maintain a stock of contraceptive methods when “they’re stocked out from the source’ or ‘we got supply from KEMSA of enough stock of combined oral contraceptive but of short expiry’ (PJM Workshops), meaning KEMSA fails to deliver family planning commodities or brings commodities that are due to expire in the near future.

Key informants also explained that some facilities obtained contraception from local suppliers, who were more efficient in delivering commodities and supplies, but also more expensive than sourcing contraception from KEMSA. Although family planning commodities were supposed to be distributed to county health facilities free of charge, the distribution was pegged to orders of other commodities from KEMSA. If no other commodities were purchased, family planning commodities would therefore not be delivered.

Additional concerns were that the county-level financing for family planning commodities was unreliable and vulnerable to pilferage:

Since 2013 when we started implementing the new constitution, the devolved system of governance, family planning has never been budgeted for. We just have to … budget for family planning and that should be a line budget for family planning. Once that is there, we will be safe, and they just make sure again that that money is not reallocated for any other function (Private Sector/NGO High-level Staff).

Some wanted those resources directed to supply chain management:

The commodity supply chain has also some major loopholes. So, you find that some of the commodities come from the KEMSA stores but whatever reaches at the sub county stores is little. The sub-county stores whatever will be given to the facilities. Some will also -- … may be -- stolen. There is pilferage. (Private Sector Facility Director).

PJM workshop participants disagreed that pilferage was the cause of orders being incompletely fulfilled, believing the cause was stockouts higher up the chain.

#### Documentation

Some key informants thought facilities experienced stockouts because providers were not completing documentation appropriately and failing to order the commodities they needed.

If healthcare providers don’t invest in reporting, then we miss it. You may provide a service but if it is not captured anywhere, documented anywhere, then basically it is like, it never happened. It is important to enhance reporting’ (Private Sector/NGO High-level Staff).

Key informants holding that attitude thought that facilities could reduce contraceptive stockouts by providing inventory management training for facility staff (Senior Government Official). However, others emphasized the difficulty of forecasting their family planning needs, not knowing if or when the order would come.

#### Redistribution

Others suggested commodity redistribution between facilities, a practice whereby facilities with a shortage of commodities can source their needs from nearby facilities with a corresponding surplus. However, this option is not without challenges, including the cost of a vehicle, gas, and drawing on an already-insufficient workforce. Some used grant funding from international NGOs for vehicles and fuel, but to achieve sustainability, recognized they needed to incorporate these expenses in their on-going budget. Another stopgap was a buffer stock to cushion the inventory during delivery delays, though this may occasionally result in some portion of the buffered stock becoming expired. Another concern about redistribution was that when providers left their clinics to obtain commodities, there could be no one available to see clients and it was ultimately counterproductive to have ‘break in service provision because of shortage of commodities’ (Private Sector/NGO High-level Staff). Proponents of commodity redistribution wanted to ‘have somebody at the top of supply chain’ (Private Sector Facility Director) to keep a bird’s eye view to make redistribution more efficient, rather than having individual facilities calling around to try to find available supply at neighboring facilities.

## Discussion

In our results, we identified the high prevalence of stockouts among a random selection of facilities that comprised 10 % of all public-sector facilities in Western Kenya. Within the mix of preferred methods found to be out of stock, the injectable was most commonly stocked out and is also the most popular method in Kenya [15]. Women and key informants from the healthcare system corroborated findings from mystery clients that stockouts are perceived as a common and important barrier to women’s uptake of desired family planning methods in the public sector. We also found a negative effect on healthcare providers, who characterized a work environment with inconsistent supply as demoralizing and an interference with community trust, which may further erode if stockouts are an occasion or excuse for solicitation of informal fees [21]. These findings are in line with other studies conducted in similar settings, which also found that contraceptive stockouts limited women’s ability to use their preferred method, especially the injection. These prior studies document that stockouts drove women to seek out contraceptives from more expensive sources, caused women to discontinue use and risk a pregnancy, and frustrated providers who felt unable to provide high quality or full range of services [4, 7, 22, 23]. Early data indicate that the COVID-19 pandemic may have had further negative impact on supply chains and stockouts in Kenya [24]. As the pandemic continues to impact supply chains around the world, collecting data on stockouts of preferred methods over time and the impact of these stockouts on patients and providers is important for ensuring contraceptive autonomy for women in Kenya.

Key informants viewed stockouts as a complex, macro policy issue. Although participants commented on strategies facilities could employ to maintain a stock despite a complicated supply chain, they emphasized the need for large-scale supply chain reform. Policy solutions for addressing contraceptive stockouts will therefore need to be multi-faceted, as informants identified multiple points in the supply chain that can culminate in facility-level stockouts. As free family planning is only available via KEMSA, addressing accurate forecasting, method availability, and prompt delivery from this state corporation is essential to decrease stockouts. Financing family planning is clearly essential to keep it free for clients at public facilities. It may be worthwhile to consider how to finance necessary supplies (including insertion supplies and pregnancy tests if those are going to continue to be required) as well as labor for supply chain management at the facility. Inventorying and ordering can represent a significant administrative burden, to which providers attributed the reduced availability to attend to patients in our analysis of provider absenteeism [25].

While policy is addressed, other solutions are also needed to more immediately reduce stockouts. In Nigeria, one study found that using midwives to deliver resupplies of contraception and collect 2 months of data on supply use was effective for forecasting, maintaining regular deliveries, and reducing clinic stockouts [26]. An intervention to address stockouts in Mozambique found positive results from monthly supply audits and material incentives for maintaining supplies, but did not find any evidence to support their hypothesis that stockouts were linked to low provider motivation to manage supplies [27]. However, increased supervision did result in better record-keeping and monitoring of stocks [27]. Increasing data collection was also successful in drastically lowering contraceptive stockouts in Senegal, where an Informed Push Model used third-party logistics providers to conduct regular facility visits, collect data, re-stock contraceptives, and create long-term forecasts for contraceptive use, although this method required client payment for methods [28]. These success stories indicate that more regular deliveries, in-person data collection, and use of data for forecasting could be successful if the supply chain can be maintained, if products are delivered well before expiry dates, and adequate staffing found.

This study’s limitations include the composition of our focus groups discussions, which largely skewed toward older women, making our results less generalizable to younger women, who may face additional barriers to contraceptive use. Another limitation is the use of mystery clients, who cannot officially verify the absence of contraceptive commodities. Mystery clients only indicate how often providers communicate to clients that there are stockouts. ‘Artificial stockouts’, where methods are available, but providers claim stockouts to avoid service delivery for another reason, may be occurring during mystery client visits. Even so, mystery client data can provide valuable insight into how often family planning clients have their choices constrained by stockouts, real or artificial. Finally, we employed a snowball sampling technique to recruit participants in our in-depth interviews, which may have resulted in similar perspectives within our sample of informants. However, the diverse perspectives reported by participants regarding stockout causes and solutions suggests our recruitment strategy did not necessarily limit our sample to those with similar viewpoints.

## Conclusion

Contraceptive stockouts impede sustained use of contraceptives in Kenya, which has dire consequences for women’s ability to use their preferred method of contraception and prevent unintended pregnancy. While the supply chain issues in Kenya are wide reaching and multi-levelled, we have identified areas where funding and sustained action could ameliorate the frequency and severity of stockouts. Further research is needed to test interventions, such as increased monitoring and contraceptive delivery and use of frequently collected data for accurate forecasting, in a Kenyan setting.

## Data Availability

The datasets used during the current study are available from the corresponding author on reasonable request.

## Abbreviations

CJM: Client Journey Map
DHS: Demographic and Health Survey
FGD: Focus Group Discussion
IUD: Intrauterine Device
KEMSA: Kenya Medical Supplies Authority
KII: Key Informant Interviews
NGO: Nongovernmental Organization
PJM: Provider Journey Map
PMA: Performance Monitoring and Accountability
